# The Correlation Between and Variability of Three Balance Scales in the Assessment of Balance Function in Patients With Ataxia

**DOI:** 10.31083/RN48265

**Published:** 2026-05-25

**Authors:** Mei Ye, Shengnan Zhang, Dan Li, Baoji Li, Zhouyao Hu

**Affiliations:** ^1^Department of Neurology, The Affiliated Hospital of Hangzhou Normal University, 311121 Hangzhou, Zhejiang, China; ^2^Translational Medicine Center, The Affiliated Hospital of Hangzhou Normal University, 311121 Hangzhou, Zhejiang, China; ^3^Department of Neurology, Hangzhou First People's Hospital Tonglu Campus (Tonglu First People's Hospital), 311500 Hangzhou, Zhejiang, China

**Keywords:** spinocerebellar ataxia, Berg Balance Scale, Semans Scale, Balance Coordination Test, ICARS

## Abstract

**Background:**

Spinocerebellar ataxia (SCA) is a rare group of hereditary degenerative disorders with major symptoms such as unsteady gait, dysarthria, and finger-nose instability. At present, the Berg Balance Scale (BBS) is a widely utilized balance assessment tool for SCA patients, exhibiting high reliability. The objective of this study was to ascertain whether the Semans Scale and the Balance Coordination Test can also be utilized for balance assessment in SCA patients.

**Methods:**

A total of 32 patients with SCA who had been diagnosed according to previously reported molecular criteria were recruited between 2021 and 2022. In addition, all patients completed assessments for all three scales.

**Results:**

The results of the study demonstrated a moderate positive correlation between the BBS and both the Semans Scale and Balance Coordination Test scores (BBS versus Semans: r = 0.568, *p* < 0.001; BBS versus Balance Coordination Test: r = 0.625, *p* < 0.001). However, the Wilcoxon signed-rank test showed statistically significant differences between BBS and both Semans Scores (Z = –2.955, *p* = 0.003) and Balance Coordination Test scores (Z = –3.666, *p* < 0.001).

**Conclusions:**

The Semans Scale and Balance Coordination Test reflect the balance function of SCA patients to a certain extent and can be used as valid complements to the BBS, providing additional information for clinical treatment and rehabilitation.

## 1. Introduction

The background of the study is as follows: spinocerebellar ataxia (SCA) is a rare group of inherited neurological disorders characterized by ataxic symptoms [1]. These disorders are typically caused by the aberrant amplification of CAG repeats in genes, which results in the aberrant aggregation of proteins and degenerative changes in nerve cells [2]. The pathogenesis of SCA remains incompletely understood. However, studies have demonstrated a strong correlation between SCA and gene mutations and aberrant protein aggregation. The principal clinical manifestations of SCA include unsteady gait, dysarthria, oculomotor disorders, and ataxia of the limbs [3]. These symptoms have a profound impact on patients’ quality of life and ability to perform daily activities [4].

Despite the absence of efficacious pharmacological treatments for SCA, prompt diagnosis and effective symptom management are crucial in enhancing patients’ quality of life. In clinical practice, the assessment of ataxia and disease progression in patients with SCA is a crucial foundation for the development of treatment and rehabilitation programs. The most common assessment instruments include the Scale for the Assessment and Rating of Ataxia (SARA), the International Cooperative Ataxia Rating Scale (ICARS), and the Berg Balance Scale (BBS) [5, 6, 7, 8]. Among these, the BBS is a standardized scale for assessing balance function that is widely used in the assessment of balance function in SCA patients due to its high reliability and validity [9]. ICARS was selected because it provides a comprehensive assessment of multiple domains of ataxia, which was relevant to the objectives of this study.

The Semans Balance Scale and Balance Coordination Scale are also commonly utilized balance assessment tools that are primarily employed to evaluate the balance abilities of patients with stroke and pediatric cerebral palsy [10]. The Semans Balance Scale is an observational assessment method that evaluates the subject’s ability to maintain balance in three positions: standing, kneeling, and two-knee kneeling [11]. Low scores indicate poor balance. Unlike the BBS, the SBS provides a more granular assessment of static postural control and stability during lower-level developmental positions, which are often compromised in SCA patients before they lose complete standing balance. The Balance Coordination Test is used to assess the subject’s balance coordination ability in various tasks and postures. Nevertheless, the utilization of these two scales in SCA patients has not been sufficiently validated, and the extent to which they can be relied on to assess balance function in SCA patients remains uncertain. While the BBS measures “whether” a task is completed, the Balance Coordination Test (BCT) places more emphasis on the smoothness and coordination of postural adjustments during limb movements.

Given these considerations, the objective of this study is to examine the feasibility of utilizing the Semans Balance Scale and Balance Coordination Test in SCA patients and assess their potential as instruments for evaluating balance function in SCA patients. The present study will analyze the correlation and variability of the ICARS, BBS, Semans Balance Scale, and Balance Coordination Test in assessing balance function in patients with SCA. To this end, these tests will be evaluated in 32 patients who have been genetically diagnosed with SCA. Furthermore, the applicability of the Semans Balance Scale and the Balance Coordination Test in patients with SCA will be validated.

## 2. Patients and Methods

### 2.1 Study Setting and Participants

A total of 32 patients with SCA who had been diagnosed according to previously reported molecular criteria were recruited from the Department of Neurology at the Affiliated Hospital of Hangzhou Normal University between 2021 and 2022. All participants have signed informed consent forms. To be included in the study, participants had to meet the following criteria: they had to have a confirmed diagnosis of SCA, and they could not have had any unspecified genotypes or disseminated phenotypes. Additionally, they had to have been able to cooperate during the research process and had been assessed using the scales. In our study, the diagnosis of spinocerebellar ataxia (SCA) was confirmed by genetic testing. The included patients were genetically confirmed cases of SCA, including SCA1, SCA2, and SCA3.

### 2.2 Data Collection and Balance Scales Assessment

The ICARS, BBS, Semans, and Balance Coordination Test were evaluated by a single assessor. The BBS was primarily designed to assess balance in patients with SCA; However, additional balance scales are also required to supplement or serve as references for BBS. The BBS was a measure of multitasking ability comprising 14 items requiring the participant to balance during a variety of tasks and in different positions, with the level of difficulty varying across items. Each item was assigned a score on a scale from 0 to 4, with a maximum score of 56. A lower score indicated an elevated risk of falling [12]. The Semans test was primarily utilized to assess a patient’s capacity to maintain equilibrium [11]. The test comprised eight items, each requiring the participant to balance in a different position. These items were grouped into a total of eight levels (0–7 scores). The BCT is a 17-item assessment designed to evaluate balance capacity and motor coordination. Each item is scored on a 0–4 scale, yielding a maximum possible score of 68. The higher the score, the better the balance function.

### 2.3 Analyses

All data were analyzed using IBM SPSS version 26.0 (IBM Corp., Armonk, NY, USA). Continuous data were assessed for normality using the Shapiro-Wilk test (**Supplementary Table 1**). Descriptive statistics (mean ± SD) were reported for normally distributed variables (ICARS, BCT, and Kinetic Function), while others were summarized as medians and interquartile ranges. The variability of the scales was operationalized and assessed using the Standard Deviation (SD) and the Coefficient of Variation (CV). To ensure objective comparison of scale results, all scale scores were converted to percentage form prior to statistical analysis. Additionally, internal consistency (Cronbach’s alpha) was used to represent the measurement stability. The internal consistency of the BBS and BCT scales was assessed using Cronbach’s alpha coefficient. Correlations were analyzed using Pearson or Spearman coefficients. For comparisons between the three balance scales (BBS, BCT, and Semans Balance (SB)), pairwise Wilcoxon signed-rank tests were performed. To account for multiple comparisons, the Bonferroni correction was applied, and a two-tailed *p* < 0.017 was considered statistically significant for these specific tests. Internal consistency was evaluated using Cronbach’s alpha, with values >0.7 indicating satisfactory reliability [13, 14].

## 3. Results

Table 1 presents the clinical data of 32 patients with SCA. The cohort comprised 16 males and 16 females, with a mean age of 39.23 ± 8.30 years and a mean disease duration of 8.22 ± 3.69 years. The mean ICARS score of the patients was 27.47 ± 9.72, which was further decomposed into the following categories: postural and gait disorders (12.38 ± 5.73), motor dysfunction (11.19 ± 4.53), speech disorders (2.41 ± 0.88), and oculomotor disorders (1.78 ± 0.87) (Table 1). The mean score for the BBS was 42.53 ± 12.86, the mean score for the Semans Balance Scale was 5.37 ± 1.43, and the mean score for the Balance Coordination Test was 39.56 ± 12.66.

**Table 1.** **Clinical data of SCA patients**.

Variables	mean ± SD
Numbers	32
Sex (Male/Female)	16/16
AAO (Age at onset)	30.72 ± 8.56
Age, years	39.23 ± 8.30
Disease Duration, years	8.22 ± 3.69
ICARS	27.47 ± 9.72
Posture and Gait Disturbance	12.38 ± 5.73
Kinetic Function	11.19 ± 4.53
Speech Disorder	2.41 ± 0.88
Oculomotor Disorders	1.78 ± 0.87
BBS	42.53 ± 12.86
Semans Balance	5.37 ± 1.43
Balance Coordination Test	39.56 ± 12.66

SCA, spinocerebellar ataxia.

**Fig. 1.**
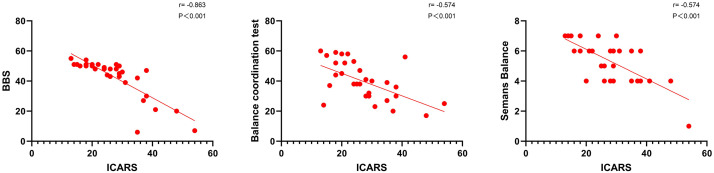
**Correlation analysis of each scale and ICARS**. The BBS, Balance Coordination Test, and Semans Scale scores were all found to be significantly negatively correlated with ICARS scores in Spearman’s correlation analysis. BBS, Berg Balance Scale; ICARS, International Cooperative Ataxia Rating Scale.

In the present study, we employed the BBS, Balance Coordination Test, and Semans Scale to assess the balance abilities of SCA patients. The results of the correlation analysis demonstrated significant correlations between the scale scores and key clinical variables, as detailed in Table 2. Specifically, the BBS, Balance Coordination Test, and Semans Scale scores were all found to be significantly negatively correlated with ICARS scores (r = –0.863, –0.574, –0.574, *p* < 0.001, respectively), Regarding postural and gait deficiencies, BBS, Balance Coordination Test, and Semans Scale scores exhibited significant negative correlations (r = –0.947, –0.613, –0.650, *p* < 0.005) (Table 2 and Fig. 1). Furthermore, the scales demonstrated high correlations in the assessment of motor function and speech disorders, though the correlations with oculomotor disorders were weaker (Table 2).

**Table 2.** **Correlation analysis of each scale and the clinical data of SCA patients**.

Variables	BBS	BCT	SB
Age	–0.108	0.030	–0.287
Disease Duration	–0.322	–0.135	–0.254
ICARS	–0.863^**^	–0.574^a**^	–0.574^**^
Posture and Gait. Disturbance	–0.947^**^	–0.613^**^	–0.650^**^
Kinetic Function	–0.660^**^	–0.517^a**^	–0.496^**^
Speech Disorder	–0.574^**^	–0.544^**^	–0.502^**^
Oculomotor Disorders	–0.289	–0.139	–0.067

SB, Semans Balance; BCT, Balance Coordination Test; ^a^, Pearson’s test; **p < 0.005.

**Fig. 2.**
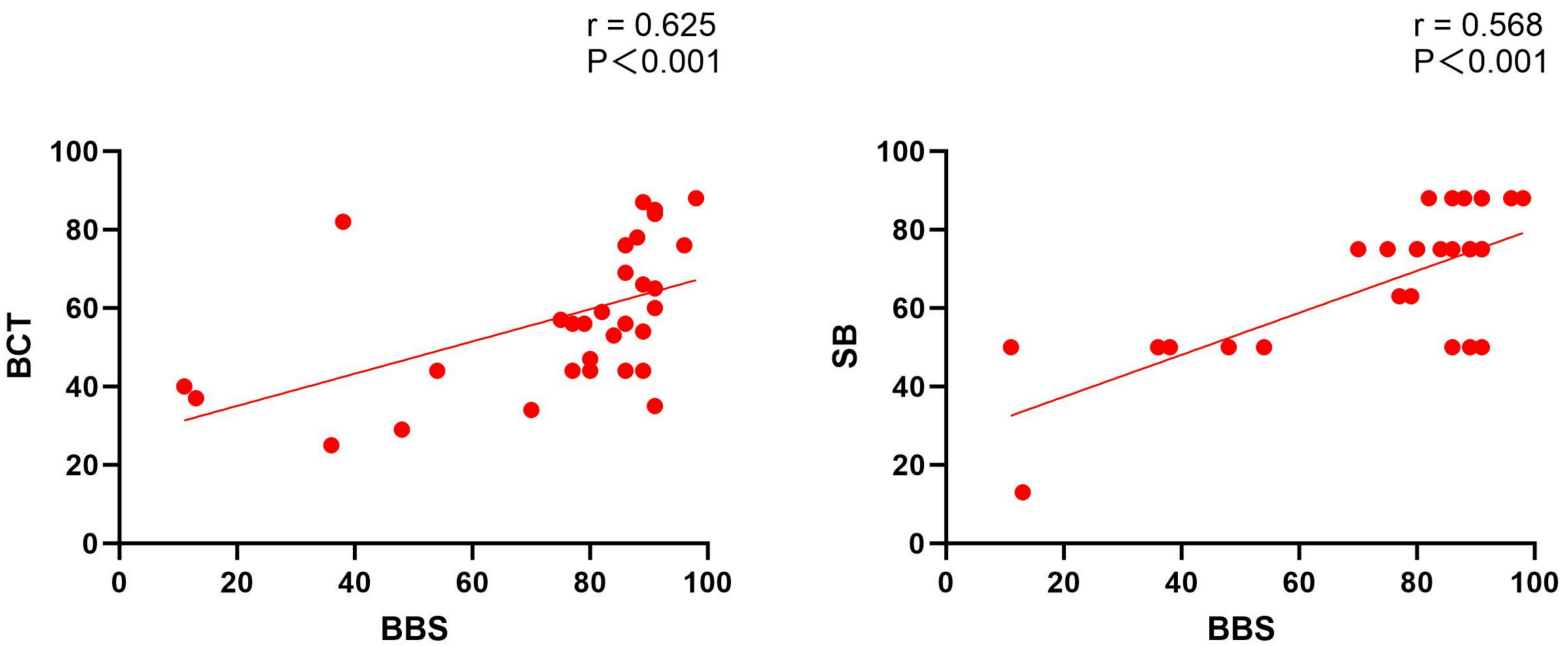
**Correlation analysis of other scales and BBS**. Spearman’s correlation analysis revealed a significant positive correlation between the BBS and both Semans Scale and Balance Coordination Test scores.

Reliability analysis showed excellent internal consistency for both instruments, with a Cronbach’s alpha of 0.946 for the BBS and 0.946 for the BCT. Regarding scale variability, the BBS scores showed a CV of 30.24%, while the BCT scores showed a CV of 32%. The high Cronbach’s alpha (0.946) further confirms the low measurement error of both instruments. Spearman’s correlation analysis revealed a moderate positive correlation between the BBS and both Semans Scale and Balance Coordination Test scores (BBS versus Semans Scale: r = 0.568, *p* < 0.001; BBS versus Balance Coordination Test: r = 0.625, *p* < 0.001) (Table 3 and Fig. 2).

**Table 3.** **Comparison between the different scales**.

Group	Median (P25, P75)	Wilcoxon signed-rank test		Spearman’s correlation test	
		*Z*	*p*	*r*	*p*
BBS	86% (76%, 90%)	–3.666	<0.001^**^	0.625	<0.001^**^
BCT	56% (44%, 76%)				
BBS	86% (76%, 90%)	–2.955	0.003^**^	0.568	<0.001^**^
SB	75% (50%, 85%)				
BCT	56% (44%, 76%)	–2.303	0.021^*^	0.372	0.036^*^
SB	75% (50%, 85%)				

*p < 0.05. **p < 0.005.

Furthermore, the Wilcoxon signed-rank test results indicated that the differences among the three scales reached statistical significance levels (BBS vs BCT: Z = –3.666, *p* < 0.001; BBS vs SB: Z = –2.955, *p* = 0.003; BCT vs SB: Z = –2.303, *p* = 0.021) (Table 3).

Furthermore, Cronbach’s alpha coefficients were calculated for the Balance Coordination Test scale. The results demonstrated that the Cronbach’s alpha coefficient was 0.946, indicating that the Balance Coordination Test scale exhibited a notably high level of internal consistency reliability. This indicates that the 17 sub-items of the Balance Coordination Test scale exhibited high consistency in scores when measuring the same underlying construct, namely the relevant symptom or function in patients with SCA.

## 4. Discussion

The results of the study indicate that while the Semans Scale and Balance Coordination Test have a degree of agreement with the BBS in terms of assessing balance in patients with SCA, they differ significantly in terms of the specific content and sensitivity of the assessment. The BBS scale is an effective tool for assessing balance dysfunction in SCA patients and provides valuable insights into the efficacy of disease treatment in this population. In this study, we evaluated the BBS, Semans Scale, and Balance Coordination Test for use with patients with SCA [10, 11]. We assessed the results using Spearman’s rank correlation and a Wilcoxon signed-rank test to determine the correlations between and differences in scores for these scales when assessing balance in SCA patients. The results demonstrated that the BBS, Balance Coordination Test, and Semans Scale exhibited significant positive correlations in terms of assessing balance ability in SCA patients. However, notable discrepancies were observed in the specific content and sensitivity of the assessments.

Spearman’s correlation analysis demonstrated significant positive correlations between BBS scores and both Balance Coordination Test and Semans Scale scores, suggesting that the Balance Coordination Test and Semans Scale reflect overlapping aspects of balance function. The BBS scale is widely utilized in patients with SCA and is considered highly reliable [15]. The BBS scale exhibited significant correlations with both the Balance Coordination Test and the Semans Scale, providing supportive evidence that these instruments are relevant to assessment of balance function in patients with SCA. Because the three scales have different scoring ranges, direct comparison of their raw scores is not statistically appropriate. Therefore, in the revised analysis, all scores were converted to percentage scores before pairwise comparison. After conversion to percentage scores, the Wilcoxon signed-rank test showed significant differences between each pair of scales, indicating that although the three instruments are correlated, their standardized score distributions are not identical and that they differ in assessment focus and sensitivity. The significant differences involving the Semans Scale may reflect its distinctive item composition and its sensitivity to certain specific aspects of balance functions. The Semans Scale was originally developed for the balance assessment in patients with stroke and pediatric cerebral palsy [11, 16]. Some of its items may not fully correspond to the specific balance deficits by SCA patients, which partly explain the observed differences in standardized scores compared with the BBS and Balance Coordination Test. Nevertheless, it’s significant in correlations with both the BBS and Balance Coordination Test, indicating that it still has some validity in assessing overall balance functioning. The Balance Coordination Test showed a high correlation with the BBS, suggesting that BCT has good consistency with the BBS in evaluation of balance functioning. However, after percentage-score transformation, the Wilcoxon signed-rank test also showed a significant difference between the Balance Coordination Test and the BBS, indicating that the two scales are related but not directly interchangeable. Overall, the Balance Coordination Test may serve as a supplementary instrument for evaluating balance function in SCA patients, particularly when a more detailed assessment of balance coordination is needed.

Furthermore, the Cronbach’s alpha coefficient for the Balance Coordination Test scale was 0.946, indicating that the internal consistency reliability of the Balance Coordination Test scale is exceptionally high. A high level of internal consistency reliability is indicated by the fact that the 17 sub-items of the Balance Coordination Test scale yield highly consistent scores when measuring the same underlying construct (i.e., the relevant symptom or function in SCA patients). This provides further evidence to support the reliability and validity of the Balance Coordination Test scale in clinical assessment. A high level of reliability indicates that the scale is stable and consistent across the SCA patient population, which is a valuable attribute for clinical assessment and research purposes. The high internal consistency reliability of the study results enhances the credibility of the findings and supports the use of the Balance Coordination Test scale for further clinical diagnosis and research. Unfortunately, there was no significant correlation between disease duration and various balance scales. Generally, as the disease course progresses, patients’ conditions tend to worsen and balance function gradually deteriorates. However, our study did not observe any correlation between these two factors. We speculate that this may be attributed to insufficient sample size.

The findings of this study indicate that a comprehensive assessment employing multiple scales may prove more beneficial in clinical practice, facilitating a comprehensive understanding of the state of balance function in SCA patients. The BBS, as a standardized scale, can provide a reliable assessment of balance ability, while the Balance Coordination Test and Semans Scale can be utilized as complementary tools to provide further insight into balance ability. This multi-scale assessment method can assist clinicians in developing effective individualized rehabilitation plans and improving treatment outcomes. Further studies are required to validate the applicability and validity of the Balance Coordination Test and Semans Scale in various patient groups, particularly in relation to various types of SCA patients, and to explore their potential value in assessing specific balance dysfunctions. Furthermore, additional research is required to ascertain the sensitivity and specificity of the scales, thus ensuring that the assessment tools can accurately reflect changes in patients’ balance abilities and provide accurate data to support clinical decision-making.

With the advancement of research, various instruments have emerged that can objectively assess the balance function of SCAs. For instance, the FOOTSCAN platform and Wearable IMUs, which are equipped with multiple sensors, can measure various data during standing or walking, such as Total Travel Way, Confidence Ellipse Area, Gait Variability, Toe-out/Toe-off Angle, etc. [17, 18]. These objective measurement data not only help differentiate between patients and healthy individuals but also hold significance for early disease identification. Future investigations should integrate these objective assessment tools alongside traditional subjective scales; such a multi-faceted approach will not only mitigate the limitations of subjective assessment data but also significantly bolster the empirical rigor and credibility of the study outcomes.

## 5. Limitation

First, the number of enrolled patients was relatively small. Due to the rarity of the disease and the high cost of genetic testing, the number of confirmed SCA patients was limited, resulting in insufficient sample size, which had a certain impact on the research findings. Additionally, healthy control subjects were not included in this study to provide normative reference data, making direct comparisons impossible. Secondly, the scale assessment did not follow the random test, which may lead to fatigue in the later scale assessment and affect the results.

## 6. Conclusions

The BBS exhibited a significant correlation with Balance Coordination Test and Semans Scale in SCA patients, despite differences in the content and sensitivity of the assessment tools employed. The Balance Coordination Test and Semans Scale are effective complementary tools for use in the assessment of balance in SCA patients. Further research is required to validate these scales.

## Availability of Data and Materials

The data that support the findings of this study are available on request from the corresponding author. The data are not publicly available due to containing information that could compromise research participant privacy.

## Supplementary Material

Supplementary material associated with this article can be found, in the online version, at https://doi.org/10.31083/RN48265.

## References

[1] Ciricugno A, Oldrati V, Cattaneo Z, Leggio M, Urgesi C, Olivito G (2024). Cerebellar Neurostimulation for Boosting Social and Affective Functions: Implications for the Rehabilitation of Hereditary Ataxia Patients. *Cerebellum (London, England)*.

[2] Klockgether T, Mariotti C, Paulson HL (2019). Spinocerebellar ataxia. *Nature Reviews. Disease Primers*.

[3] Sullivan R, Yau WY, O’Connor E, Houlden H (2019). Spinocerebellar ataxia: an update. *Journal of Neurology*.

[4] Buchholz M, Weber N, Rädke A, Faber J, Schmitz-Hübsch T, Jacobi H (2024). Health-Related Quality of Life in Patients with Spinocerebellar Ataxia: a Validation Study of the EQ-5D-3L. *Cerebellum (London, England)*.

[5] Amarante TRP, Takeda SYM, Teive HAG, Zonta MB (2017). Impact of disease duration on functional status of patients with spinocerebellar ataxia type 2. *Arquivos De Neuro-psiquiatria*.

[6] Konno KM, Zonta MB, Guimarães ATB, Camargo CHF, Munhoz RP, Raskin S (2021). Balance and physical functioning in Spinocerebellar ataxias 3 and 10. *Acta Neurologica Scandinavica*.

[7] Schmitz-Hübsch T, du Montcel ST, Baliko L, Berciano J, Boesch S, Depondt C (2006). Scale for the assessment and rating of ataxia: development of a new clinical scale. *Neurology*.

[8] Trouillas P, Takayanagi T, Hallett M, Currier RD, Subramony SH, Wessel K (1997). International Cooperative Ataxia Rating Scale for pharmacological assessment of the cerebellar syndrome. The Ataxia Neuropharmacology Committee of the World Federation of Neurology. *Journal of the Neurological Sciences*.

[9] Miyata K, Kondo Y, Bando K, Hara T, Takahashi Y (2024). Structural Validity of the Mini-Balance Evaluation Systems Test in Individuals With Spinocerebellar Ataxia: A Rasch Analysis Study. *Archives of Physical Medicine and Rehabilitation*.

[10] Yulong B, Yi W, Yongshan H, Deheng C, Limin S, Xiaoli D (2003). Rehabilitation assessment and treatment of cerebellar ataxia patients. *China Clinical Rehabilitation*.

[11] Bian R, Huo M, Liu W, Mansouri N, Tanglay O, Young I (2023). Connectomics underlying motor functional outcomes in the acute period following stroke. *Frontiers in Aging Neuroscience*.

[12] Santos de Oliveira LA, Martins CP, Horsczaruk CHR, Lima da Silva DC, Martins JVP, Vasconcelos LFR (2015). Decreasing fall risk in spinocerebellar ataxia. *Journal of Physical Therapy Science*.

[13] Cronbach LJ (1951). Coefficient alpha and the internal structure of tests. *Psychometrika*.

[14] Bland JM, Altman DG (1997). Cronbach’s alpha. *BMJ (Clinical Research Ed.)*.

[15] Milne SC, Roberts M, Ross HL, Robinson A, Grove K, Modderman G (2023). Interrater Reliability of the Scale for the Assessment and Rating of Ataxia, Berg Balance Scale, and Functional Independence Measure Motor Domain in Individuals With Hereditary Cerebellar Ataxia. *Archives of Physical Medicine and Rehabilitation*.

[16] Wang YL (2018). *Functional Assessment of Rehabilitation*.

[17] Velázquez-Pérez L, Rodriguez-Labrada R, González-Garcés Y, Arrufat-Pie E, Torres-Vega R, Medrano-Montero J (2021). Prodromal Spinocerebellar Ataxia Type 2 Subjects Have Quantifiable Gait and Postural Sway Deficits. *Movement Disorders: Official Journal of the Movement Disorder Society*.

[18] Schwabova J, Zahalka F, Komarek V, Maly T, Hrasky P, Gryc T (2012). Uses of the postural stability test for differential diagnosis of hereditary ataxias. *Journal of the Neurological Sciences*.

