# Supplementary material for: The Correlation Between and Variability of Three Balance Scales in the Assessment of Balance Function in Patients With Ataxia
Source: Rev Neurol. 2026 May 25;81(5):48265. doi: 10.31083/RN48265 (PMC13221673; doi:10.31083/RN48265)
Supplement: Supplementary file 1 [file 1576-6578-81-5-48265-s1.zip › Supplementary Material.docx]

**Supplementary Table 1. Normality Test of Clinical Data in SCA Patients**

| **Variables** | **Shapiro-Wilk test** | **P** |
| --- | --- | --- |
| **Age, years** | 0.901 | 0.007 |
| **Disease Duration, years** | 0.906 | 0.009 |
| **ICARS** | 0.951 | 0.156 |
| Posture and Gait Disturbance | 0.900 | 0.006 |
| Kinetic Function | 0.938 | 0.064 |
| Speech Disorder | 0.737 | ＜0.001 |
| Oculomotor Disorders | 0.857 | ＜0.001 |
| **BBS** | 0.740 | ＜0.001 |
| **Semans Balance** | 0.848 | ＜0.001 |
| **Balance Coordination Scale** | 0.948 | 0.124 |
